# WHICH LESIONS ARE AT HIGHER RISK OF DEVELOPING COLORECTAL CARCINOMAS: SUPERFICIALLY ELEVATED SERRATED LESIONS OR DEPRESSED LESIONS?

**DOI:** 10.1590/0102-672020220002e1716

**Published:** 2023-01-09

**Authors:** Artur Adolfo PARADA, Filadelfio Euclydes VENCO, Miguel Reynaldo VARCA-NETO, Roberto EL IBRAHIM, Paula Bechara POLETTI, Helcio Pedrosa BRITO, Heloisa de Fátima SARE, Osvaldo MALAFAIA

**Affiliations:** 1 Faculdade Evangélica Mackenzie do Paraná, Postgraduate Program in Principles of Surgery – Curitiba (PR), Brazil;; 2 Nove de Julho Hospital, Center for Endoscopic Diagnosis and Therapeutics of São Paulo – São Paulo (SP), Brazil;; 3 Evangélico Mackenzie University Hospital – Curitiba (PR), Brazil.

**Keywords:** Colonoscopy, Mass Screening, Colorectal Neoplasms, Colon, Polyps, Colonoscopia, Programas de Rastreamento, Neoplasias, Colo, Pólipos

## Abstract

**BACKGROUND:**

There are lesions that are still being missed in colonoscopy. Many of those could be superficially elevated serrated lesions or depressed ones.

**AIMS:**

The aim of this study was to compare the histopathological characteristics of these lesions and their risks for submucosal carcinoma.

**METHODS:**

This is a retrospective, cross-sectional, and observational study comparing 217 superficially elevated serrated lesions larger than 5 mm resected by colonoscopies (G1) with 558 depressed lesions (G2).

**RESULTS:**

In G1, 217 lesions were found in 12,653 (1.7%) colonoscopies; in G2, 558 lesions were found in 36,174 (1.5%) colonoscopies. In G1, 63.4% were women and in G2, there was no gender predominance. The average size of G1 was 16.2 mm and G2 was 9.2 mm (p<0.001). G1 predominated on the proximal colon and G2 on the distal and rectum (p<0.001). In G1, there were 214 (98.6%) low-grade intramucosal neoplasia and 3 (1.4%) high-grade intramucosal neoplasia. Excluding 126 hyperplastic polyps and considering 91 sessile serrated adenomas in G1, we observed 88 (96.7%) low-grade intramucosal neoplasia and 3 (3.3%) high-grade intramucosal neoplasia; in G2, we observed 417 (74.7%) low-grade intramucosal neoplasia, 113 (20.3%) high-grade intramucosal neoplasia, and 28 (5.0%) submucosal adenocarcinomas (p<0.001).

**CONCLUSION:**

Depressed lesions significantly had more high-grade intramucosal neoplasia and more invasive carcinomas in the submucosal layer than superficially elevated serrated lesions and more than superficially elevated sessile serrated adenomas.

## RESUMO

**RACIONAL:**

Há ainda lesões que podem ser perdidas nas colonoscopias. Muitas delas poderiam ser serrilhadas superficialmente elevadas ou deprimidas.

**OBJETIVO:**

Comparar as características histopatológicas destas lesões e seus riscos para carcinoma invasivo para a submucosa.

**MÉTODO:**

Estudo retrospectivo, transversal, observacional comparando 217 lesões serrilhadas superficialmente elevadas com mais de 5 mm e ressecadas por colonoscopias (G1) com 558 lesões deprimidas (G2).

**RESULTADOS:**

As 217 lesões do G1 foram encontradas em 12.653 colonoscopias (1,7%) enquanto as 558 do G2 ocorreram dentre 36.174 colonoscopias (1,5%). No G1, 63,4% eram mulheres e no G2 não houve predominância de gênero. O tamanho médio foi no G1, 16,2 mm e no G2, 9,2 mm (p<0,001). G1 predominaram no cólon proximal e G2, no distal e reto (p<0,001). No G1, ocorreram 214 (98,6%) neoplasias mucosas de baixo grau e três de alto grau (1,4%). Excluídos 126 pólipos hiperplásicos e considerados os 91 adenomas sésseis serrilhados, no G1 observou- se 88 (96,7%) neoplasias mucosas de baixo grau e três (3,3%) de alto grau, e no G2, 417 (74,7%) neoplasias mucosas de baixo grau 113 (20,3%) de alto grau e 28 (5,0%) adenocarcinomas invadindo a submucosa (p<0,001).

**CONCLUSÕES:**

As lesões deprimidas apresentaram significativamente mais neoplasias mucosas de alto grau e carcinomas invasivos para a submucosa do que as serrilhadas superficialmente elevadas e mais do que os adenomas sésseis serrilhados superficialmente elevados.

## INTRODUCTION

Colonoscopy has been widely used as a diagnostic test and in colorectal cancer screening programs. Even with high-quality and thorough colonoscopies, there is a loss of lesions, mainly nonpolypoid or superficial ones, and in the proximal colon. Superficially elevated and adenomatous lesions are relatively frequent and are more difficult to diagnose than polypoid ones; however, due to their greater vascularization and surface changes, they are not as difficult as depressed lesions and the so-called superficially elevated serrated lesions (hyperplastic or sessile serrated adenomas). These are often presented as superficially elevated lesions, larger than 5 mm, as swollen or redundant folds, often covered by mucus, with a cloudy appearance, ill-defined borders, the same color as the adjacent mucosa, and small dilated and spiral vessels^1,7,9,13^, which we call the “Batman injury.” The small dark spots and the open stellar crypt pattern (type IIO), visualized with narrow band image (NBI) or with dyes and with high-resolution or image magnification devices, seem to indicate dilatations of the crypt holes above the mucosal muscle layer on histological examination of sessile serrated adenomas^21^. The surface pattern and vascular pattern are used in the most modern classifications of colorectal lesions, such as the Japanese NBI Expert Team (JNET Classification), in which superficially elevated serrated lesions, without dysplasia or cancer, are classified as type I^28^.

Many studies suggest that interval carcinomas, that is, those diagnosed before a colonoscopy is performed within the time frame recommended in the guidelines (generally between 3 and 5 years), are more proximal than distal and are likely to be undiagnosed, among others. As they develop from superficial lesions or lesions that have not been adequately resected^10,21,25,26^, these missed lesions can be superficially elevated (IIA), superficially flat (IIB), such as serrated ones, or superficially depressed (IIC, IIC+IIA, or IIA+IIC)^15,20^.

Currently, serrated lesions are subdivided by the World Health Organization into hyperplastic polyps, sessile serrated adenomas, traditional serrated adenomas, and mixed polyps. These subtypes are generally identified by architectural and cytological features, location in the colon, and the extent and location of the proliferative zone. Virtually all superficially elevated and serrated lesions are hyperplastic or sessile serrated adenomas^25,30^.

In recent years, serrated lesions have come to be considered as one of the important pathways of colorectal carcinogenesis and may represent, according to several authors, from 6.0 to 30% of all colorectal carcinomas; however, the percentage of each pathway in this carcinogenesis is not yet known for certain^2,12,25^. However, they have the potential, yet to be defined, to evolve into invasive cancer, with a molecular profile different from each other and from carcinomas in conventional adenomas^2,6,7,25,31,32^. A high risk of cancer in sessile serrated adenomas is reported in patients with serrated polyposis syndrome^13^ and in the elderly with sessile or protruding components associated with it^18,21^.

In clinical practice, serrated lesions above the sigmoid or more proximal, greater than 5 mm, should be resected and can be considered sessile serrated adenomas even if they have been reported as hyperplastic polyps^1,5,13,25,29^.

On the contrary, recognizing depressed lesions during colonoscopies is very important because they can be invasive even when they are very small. We need to pay attention to small areas with color change (a little reddish, some pale, or discolored), small bleeding points, interruptions of the capillary network, or small local deformations. The use of dyes or high-resolution devices with digital chromoscopies is mandatory. In Japan, they represent 2.3% of all polypoid and nonpolypoid lesions of the colon and rectum and 5.5% of superficial lesions^15^. Despite the relatively low prevalence of polypoid and nonpolypoid lesions (67.4, 29.9, and 2.7%), they represented 71.5% of 249 carcinomas with submucosal invasion^20^.

With the use of large-scale colonoscopy and the resection of lesions, early cancers of the colon and rectum were increasingly diagnosed. The smaller and more superficial the lesions we diagnose and resect, the closer we get to understanding the histogenesis of colorectal cancer. The superficial adenomatous and small ones present very low rates of invasive malignant lesions to the submucosa^15^. Superficial lesions, especially the serrated ones that are superficially elevated and depressed, are probably those that are mostly unnoticed when performing colonoscopies. The analysis of the characteristics of these lesions can generate interesting results obtained from the comparison of the two groups in relation to our understanding of the evolution of the diagnosed cytological and structural alterations and, in a large series, provide subsidies for decision-making in resection procedures and for guidelines in screening programs aimed at reducing the number of missed lesions and consequently reducing interval carcinomas.

The objective of this study was to compare the histopathological characteristics of a sample of superficially elevated lesions, larger than 5 mm, that were completely resected by colonoscopies and histologically diagnosed as serrated (hyperplastic and sessile serrated adenomas) with another sample of depressed neoplastic lesions, of any size, that were also resected by colonoscopies and their risk of progression to invasive submucosal cancer.

## METHODS

This study was approved by the Ethics Committee of the Medical Research Institute, Mackenzie Evangelical Faculty of Paraná, Curitiba, PR, Brazil, under number CAAE 1,433,052. This is a horizontal cross-sectional retrospective study that aimed to analyze a group of patients undergoing endoscopic resection of superficially elevated lesions with a diameter of 5 mm or more and with the histopathological diagnosis of serrated lesion (G1), compared with another group with the diagnosis of depressed lesions, also resected by colonoscopies (G2).

Superficially elevated lesions greater than 5 mm with the histopathological diagnosis of serrated lesions in colonoscopy resections performed between January 2012 and May 2019 in 12,653 examinations (G1) and superficially depressed neoplastic lesions resected in 36,174 examinations (G2) were evaluated. Patients were selected from the database of the Gastrointestinal Endoscopy Service of Hospital 9 de Julho, in São Paulo, SP, Brazil, from January 2006 to May 2019, where 15 doctors specialized in digestive endoscopy and 4 pathologists dedicated to the gastrointestinal area worked.

The examinations were performed with Olympus, Pentax, or Fujinon brand colonoscopies after routine preparation of the colon with a liquid diet and laxatives the day before and 1000 ml of 10% mannitol solution, orally, 6–8 h before the examination. Dyes such as 0.4% indigo carmine or 3% acetic acid or digital chromoscopy, with or without image magnification, were used. Sedation was performed by anesthesiologists. The cecum, ascending, and transverse colon were considered proximal locations, and the splenic flexure, descending colon, sigmoid, and rectum were considered distal.

The smaller lesions were resected with cold loops or by monoblock mucosectomies up to 2 cm in diameter, and the larger ones by mucosectomies in fragments, by the technique underwater (submarine), or, in a few larger cases, by the hybrid technique or by endoscopic dissection of the submucosa. Cases in which the lesions did not elevate (no elevation sign) after injection of saline or hypertonic solution into the submucosa, with endoscopic signs suggestive of deep submucosal infiltration, with many technical difficulties, very large and circumferential lesions or in very difficult locations, were excluded and referred for surgical treatment. Elevated or polypoid lesions, polyposis, inflammatory bowel diseases, or when the lesion was not recovered for histological examination were also excluded.

The pieces were stretched on Styrofoam or cardboard with needles, fixed in 10% formalin, then cut every 2 mm, examined microscopically after staining with H&E, and evaluated the type of histological lesion and the lateral and vertical margins. Submucosal invasion was subdivided into three levels: sm1, sm2, and sm3. The limit of this measurement, which considered patients to be practically cured with minimal risk of lymph node metastases, was 1000 μM (sm1)^15^.

The lesions were classified according to a macroscopic appearance by the Paris Classification, slightly modified^17^; the Japanese school used this method to categorize lateral spreading tumor (LST)^32^. Superficially raised serrations were included in G1 and nongranular pseudodepressed lateral spreading lesions (LST-NG-PD) were considered depressed lesions or were classified as mixed lesions: superficially depressed with minor superficially elevated areas (IIC+IIA) or superficially elevated with areas depressed minors (IIA+IIC) and included in G2, to compare with the large serrated superficial lesions.

**Figure 1.**
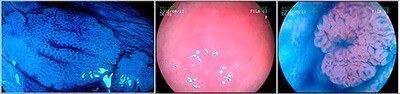
Hyperplastic lesions: (A) superficially elevated hyperplastic lesion – image magnification and indigo carmine; (B) pale hyperplastic lesion, IIA/IIB, with adjacent edema, measuring 3 mm and with ill-defined edges on conventional examination; and (C) stellar crypt pattern with image magnification and indigo carmine.

**Figure 2.**
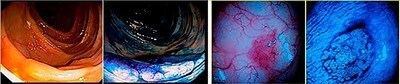
Superficially elevated serrated adenomas: (A) superficially elevated serrated lesion – finely granular, 3.0 cm long – in the ascending colon; (B) same lesion with indigo carmine; (C) type IIA/IIB lesion, highly vascularized, 5 mm in diameter; and (D) same lesion after indigo carmine and image magnification showing a stellar-like crypt pattern. Anatomopathological: Low-grade intraepithelial neoplasia – serrated.

For histology, the Vienna Classification^8^ was used; serrated lesions were classified according to the WHO classification^25,30^; hyperplastic lesions larger than 5 mm and in any location were considered a subgroup of superficially elevated serrated lesions, together with the subgroup of sessile serrated adenomas (Figures 1 and 2).

In hyperplastic polyps, all crypts should show normal maturation toward the surface, with a sawtooth pattern in the upper third of the crypts and with the basal proliferative zone normally located. In the sessile serrated adenomas, one or more crypts showed distorted growth, with dilatation of their basal portion, with the shape of a boot, capital letter L, or anchor. Criteria are variable, but only a clearly abnormal crypt is sufficient for the diagnosis of sessile serrated adenomas^10,25,30^. They were recognized by their architectural and (secondarily) cytological changes, with or without mild dysplasia (more stained and elongated nuclei, pseudostratification, apoptosis, increased mitotic activity, and mucin loss, as seen in conventional adenomas), and were considered serrated low-grade intraepithelial or mucosal neoplasms (NMBG). Those with intense architectural alterations or with intense cytological dysplasia were considered serrated intraepithelial or mucosal neoplasms (NMAG), characterized by greater nuclear enlargement, loss of polarity, and accompanying architectural complexity. Only cases with submucosal invasion were considered carcinomas^18^.

**Figure 3.**
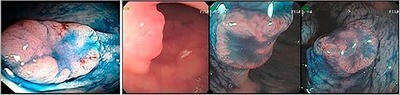
Depressed lesions – intramucosal and adenocarcinoma: (A) IIA+IIC lesion in the descending colon – high-grade intramucosal neoplasm; (B) IIC+IIA lesion seen in profile and simulating a sessile sigmoid lesion; (C and D) same lesion seen from the front and classified as IIC+IIA, 1.2 cm in diameter – carcinoma infiltrating up to sm1.

Depressed lesions were also classified as low-grade intraepithelial or mucosal neoplasms (LMBG) or high-grade intraepithelial or mucosal neoplasms (MAGN) and as carcinomas when submucosal invasions occurred. Lesions with intense intraepithelial or mucosal dysplasia, with or without carcinomas with submucosal invasion, were considered high-grade neoplasms (NAG), for the purpose of statistical comparison between the two groups (Figure 3).

### Statistical analysis

To assess the association between two categorical variables, the Fisher’s exact test or the chi-square test was used. Value of p<0.05 indicated statistical significance. Data were analyzed using the computer program IBM SPSS Statistics version 20.0 (IBM Corp, Armonk, NY, USA).

## RESULTS

In G1, we had 202 patients with 217 lesions, with a mean age of 63±10.6 years (28–86). In G2, 502 patients with 558 cases had a mean age of 61.1±11.2 years (29–88). When comparing the percentage of prevalence in the age groups, between the two groups, up to 50 years old, 10.9% were serrated lesions and 6.0% depressed; between 51 and 60 years, 23.8% were serrated and 31.7% depressed; between 61 and 70 years, 43.6% were serrated and 36.5% depressed; and over 70 years, 21.8% were serrated and 25.9% depressed.

As for gender, women represented 63.4% in serrated and 48.4% in depressed (p<0.001). As for the macroscopic type, 170 LST were serrated with more than 1 cm (78.4%) and 47 IIA (21.6%); among the depressed, 131 IIC (23.5%), 71 IIC+IIA (12.7%), and 356 IIA+IIC (63.8%). As for size, 47 serrations with less than 10 mm (21.8% of 217), 138 (63.4%) with 11–20 mm, and 32 (14.8%) with more than 21 mm; among depressed patients, 446 (79.9%) were less than 10 mm, 44 (7.9%) were between 11 and 20 mm, and 12.2% were more than 21 mm. The mean size was 16.2 mm in 217 superficial serrated lesions and 9.2 mm in 558 depressed serrated lesions. There was a significant difference between the two groups in terms of size, with a greater predominance of larger lesions in G1 (p<0.0001).

Regarding the location, 159 (73.3%) serrated lesions were proximal and 58 (26.7%) distal. For depressed lesions, 42.9% were proximal (239 lesions) and 57.1% were distal (319 lesions). These differences were significant (p<0.001).

Regarding the histopathological aspect, G1, with 217 lesions, had 126 (58%) hyperplastic lesions and 91 (42%) sessile serrated adenomas. Of these, 88 (40.6%) had low-grade mucosal neoplasms, with or without mild cytological dysplasia, and 3 had severe architectural or cytological alterations, which corresponded to 1.4% of 217 lesions. Among the depressed lesions, 417 (74.8%) had low-grade mucosal neoplasms, 113 (20.2%) high-grade mucosal neoplasms, and 28 (5.0%) invasive submucosal carcinomas (Table 1).

**Table 1.** Serrated and depressed lesions: anatomopathological analysis.

Serrated	n	%	Depressed	n	%
Hyperplastic	126	58.0	Hyperplastic	0	0
SSA - NMBG	88	40.6	NMBG	417	74.8
SSA - NMAG	3	1.4	NMAG	113	20.2
CA-sm	0	0	CA-sm	28	5.0
**Total**	**217**	**100**	**Total**	**558**	**100**

SSA: serrated sessile adenomas; NMBG: low-grade mucosal neoplasm; NMAG: high-grade mucosal neoplasm; CA-sm: invasive carcinoma to the submucosa.

When we compared only the 91 sessile serrated adenomas (SSA) with 558 depressed lesions in terms of NMAG and NAG (considering together high-grade mucosal neoplasms and invasive submucosal carcinomas), we observed 88 (96.7%) in G1 with NMAG and 3 (3.3%) with NAG; in the depressed, 417 NMBG (74.7%) and 141 NAG. This difference was significant between these groups (p<0.001).

When we analyzed the relationship between lesion size and pathological findings between G1 and G2, there was a predominance of larger sizes in serrated ones (p<0.0001). Only 3 (1.4%) of 217 were sessile serrated adenomas with intense dysplasia (NMAG) and of these, 2 were in lesions with 15 mm and 1 with 25 mm. All less than 10 mm were hyperplastic or serrated sessile adenomas with or without low-grade cytological dysplasia. In depressed patients, there were NMAG and CA-sm in 19.9% with less than 10 mm, 43.2% between 11 and 20 mm, and 48.5% with more than 21 mm. In total, 141 (25.3%) of 558 lesions were NAG.

Considering the total series of serrated (G1) and depressed (G2) lesions, it was evident that submucosal carcinomas occurred significantly more in depressed lesions (p=0.0001) (Table 2).

**Table 2.** Carcinoma-sm in serrated and depressed lesions.

Lesions	Serrated	Depressed	Total
Without carcinoma	217	530	747
With carcinoma	0	28	28
Total	217	558	775

sm: submucosa (p=0.0001).

When considering SSA and depressed lesions, the presence of carcinoma invading the submucosa was also significantly higher in depressed lesions (p=0.0231) (Table 3).

**Table 3.** Carcinoma-sm – serrated sessile adenomas and depressed lesions.

Lesions	SSA	Depressed	Total
Without carcinoma	91	530	621
With carcinoma	0	28	28
Total	91	558	649

sm: submucosa; SSA: serrated sessile adenomas (p=0.0231).

## DISCUSSION

There are large series of articles published in the literature on polypoid and superficial lesions. These are the main causes of missed lesions and interval carcinomas. There are, however, a few reported cases of superficially elevated serrated and depressed lesions, which are the most difficult to diagnose at colonoscopies and may be responsible for a large number of interval carcinomas.

We did not find any work in the literature specifically comparing superficially elevated serrated lesions with depressed ones, which are very different in their histogenesis, presentations, and evolution. For many years, all lesions have been resected by colonoscopy, except for small hyperplastic polyps of the rectum and distal sigmoid, but no long-term follow-up was found in the literature; so, it is not possible to clearly assure which percentage will progress to infiltrative submucosal carcinomas. Hence, it is important to analyze a large number of these resected lesions in their early stages.

There are endoscopic difficulties in the proper diagnosis and the degree of dysplasia of serrated lesions since they are different lesions of the adenoma-cancer sequence. In these lesions, dysplasia or carcinomas are more frequently diagnosed in large pediculated or (sub)pediculated lesions, with double elevation (two layers), depressions, and reddish color^21^, and in foci of adenomas that appear on their surfaces^33^. In cases with invasive carcinomas, all had irregular or effaced crypt patterns^21,22^. However, histopathological examination of the entire lesion remains the diagnostic gold standard.

Although they are different lesions and there may be criticisms regarding the criteria we adopted, the comparison between groups considering carcinomas only when there is an invasion of the submucosa is an objective criterion.

In G1, we observed a prevalence of superficially elevated serrated lesions in 1.7% of colonoscopies. The literature shows a variation from 0 to 16.2% in the detection of serrated lesions of 5 mm and located in the transverse, ascending, and cecum^29^ or proximal to the sigmoid colon^1,13^. Regarding gender and location, our data are similar to those published in the literature^5,18^. The average size was 16.2 mm, above those reported by some published papers^5,11^.

Regarding histology, the WHO classification describes two patterns of dysplasia in SSA: serrated and conventional. In a recent study with immunohistochemistry, four dysplasia types were evidenced as follows: with minimal deviation (19%); serrated (12%); adenomatous (8%); and nonspecified (79%). However, the final conclusion of this research is that the diagnosis practically does not change^19^. Other publications report that immunohistochemistry still needs to be improved for serrated lesions^3,6,14,24^, as well as the use of molecular investigations^3,23,24^.

Our data on severe dysplasia and invasive submucosal carcinomas are similar to those published in the literature, where the percentages of these lesions were very low or not observed. One study published 0.1% of submucosal carcinoma in a hyperplastic lesion, 0.7% of high-grade dysplasia, and 0.2% of carcinoma invading the submucosa in SSA^4^. In another publication, mucosal carcinomas (high-grade mucosal neoplasms or high-grade dysplasia) were described in 13.6% of SSA and 0% of hyperplastic polyps, but carcinomas invading the submucosa were not described^11^. In an analysis of a large number of carcinomas invading the submucosa, 6% were serrated with the invasion of the submucosa and four SSA with the invasion of the submucosa (1.6% of the total of carcinomas with invasion of the submucosa)^12^. In another publication, invasive carcinomas were diagnosed in 1.3% of resected hyperplastic polyps (one case, already invading the muscularis propria) and 5.9% of SSA (four cases invading the submucosa and one the muscularis propria)^22^.

According to several authors, categorizing these serrated lesions as low-grade dysplasia can send the wrong message to clinicians. It is recommended to emphasize the nature of the advanced lesion and the need for short-term endoscopic follow-up to verify that the lesion has been completely resected. It is not yet clear that the size of 10 mm, used to define adenomas as advanced, is also applicable for sessile serrated adenomas^25^.

The mean age in G1 was 63 years, and in patients with SSA with high-grade dysplasia it was 74.3 years, but there were only three cases. In a publication in this regard, the mean age of patients with SSA was 61 years. Low-grade dysplasia occurred in 12% of cases, with a mean age of 66 years; high-grade dysplasia in 2%, with a mean age of 72 years; and invasive carcinomas related to sessile serrated adenomas in 1%, with a mean age of 76 years, suggesting that invasive submucosal carcinomas in SSA appear in older people and evolve slowly^18^.

In a recent publication, however, clinical and molecular aspects of a large number of SSA with regions of dysplasia or carcinoma were analyzed and found to be predominantly small (<10 mm), in the proximal colon and in the elderly (mean of 76.7 years). Cases with dysplasia occurred in patients with ages similar to those with carcinomas, suggesting rapid transformation to malignancy^2^.

In our series, we did not find any invasive adenocarcinoma of the submucosa in 217 serrated, superficially elevated lesions greater than 5 mm, even with 170 lesions greater than 10 mm, and of these, 32 were greater than 2 cm, which suggests that this is an infrequent event or that it occurs in smaller lesions that rapidly progress to a deep invasion of the submucosa, preventing resection by colonoscopy.

In G2, we observed a prevalence of depressed lesions in 1.5% of the colonoscopies. As for gender, the distribution was similar in males and females. In the Japanese literature, a slightly higher prevalence was reported, ranging from 2.3 to 2.7%, with a predominance in men^16,20^.

In our series, depressed lesions predominated in the distal colon and rectum in relation to the proximal colon (p<0.001), but they were present in significant percentages in the rectum and in all segments of the colon, and these data are similar to those published in the literature^15,27^.

Regarding size, the mean of depressed lesions was 9.2 mm. The mean of low-grade depressed mucosal neoplasms was 8.9 mm, of high-grade mucosal neoplasms 12.3 mm, and of submucosal carcinomas 12.2 mm, suggesting rapid evolution of the lesions. The mean age of patients with depressed lesions was 61.1 years, 61.2 years in those with NMBG, 60.2 years in those with NMAG; and 63.5 years in those with CA-sm. These data also suggest a rapid evolution of these lesions to invasive carcinomas in a younger age group than the serrated ones.

NMBG was diagnosed in 74.7% of the lesions in G2, NMAG in 20.2%, and carcinomas with submucosal invasion in 5.0%. Considering NMAG and CA-sm together, they corresponded to 25.2% of the total depressed lesions. These data are well below the Japanese series, which showed 61.2% of high-grade neoplasia in the mucosa or invading the submucosa in depressed lesions^20^ and 10.7% of submucosal invasion in lesions less than 5 mm, 52.6% with 6 at 10 mm, and 92.3% from 11 to 15 mm^16^. These data mean that there are divergences in the macroscopic classification of depressed lesions and that we probably include many adenomas with depressions, just as we include LST-PD.

An important consideration is that in the initial phase of some depressed lesions, areas with stellar crypt patterns are shown, considering these lesions as depressed with adjacent hyperplastic reactions; however, they require more detailed studies as they may eventually be depressed serrated lesions that can evolve rapidly.

When we compared the two groups with each other, we showed that women predominated in G1 and there was no difference in gender in G2 (p<0.001). As for the size, the serrated ones measured an average of 16.2 mm and the depressed ones 9.2 mm. Most serrated lesions were greater than 11 mm and most depressed had less than 10 mm (p<0.001). The serrated lesions were located more in the proximal colon and the depressed ones were in the distal colon and rectum (p<0.001); however, these ones are more evenly distributed throughout the colon and rectum.

Regarding histology, in G1, there were 214 (98.6%) low-grade mucosal neoplasms and 3 (1.4%) high-grade mucosal neoplasms. In G2, there were 417 (74.7%) low-grade mucosal neoplasms, 113 (20.3%) high-grade mucosal neoplasms, and 28 (5.0%) adenocarcinomas invading the submucosa (p<0.001). Regarding submucosal invasive carcinomas, a significant difference was observed, with a higher number of depressed lesions than serrated (p=0.0001). When we excluded 126 hyperplastic polyps from G1 and compared the 91 SSA with the 558 depressed lesions, we also observed significantly more high-grade mucosal neoplasms and submucosal invasive carcinomas in the depressed lesions (p=0.0231).

Our data indicate that depressed lesions (G2) have a higher risk of progression to high-grade mucosal neoplasms and submucosal invasive carcinomas than superficially raised serrated lesions and that they may be the main causes of interval carcinomas. In serrated lesions, an increasing gradient of architectural and cytological alterations from the rectum to the cecum was observed, mainly in the proximal colon, where it could have greater participation in cases of interval carcinomas.

## CONCLUSIONS

The depressed lesions had significantly more high-grade mucosal neoplasms and submucosal invasive carcinomas than the superficially elevated serrated ones and also more than the superficially elevated sessile serrated adenomas when considered separately.
